# Phase synchronization of delta and theta oscillations increase during the detection of relevant lexical information

**DOI:** 10.3389/fpsyg.2013.00308

**Published:** 2013-06-18

**Authors:** Enzo Brunetti, Pedro E. Maldonado, Francisco Aboitiz

**Affiliations:** ^1^Departamento de Psiquiatría, Facultad de Medicina, Centro Interdisciplinario de Neurociencia, Pontificia Universidad Católica de ChileSantiago, Chile; ^2^Programa de Fisiología y Biofísica, Instituto de Ciencias Biomédicas, Facultad de Medicina, Universidad de ChileSantiago, Chile; ^3^Center for the Neuroscience of Memory, Iniciativa Cientifica MilenioSantiago, Chile

**Keywords:** language, semantic analysis, relevance, oscillations, phase synchronization, theta, delta, gamma

## Abstract

During monitoring of the discourse, the detection of the relevance of incoming lexical information could be critical for its incorporation to update mental representations in memory. Because, in these situations, the relevance for lexical information is defined by abstract rules that are maintained in memory, a central aspect to elucidate is how an abstract level of knowledge maintained in mind mediates the detection of the lower-level semantic information. In the present study, we propose that neuronal oscillations participate in the detection of relevant lexical information, based on “kept in mind” rules deriving from more abstract semantic information. We tested our hypothesis using an experimental paradigm that restricted the detection of relevance to inferences based on explicit information, thus controlling for ambiguities derived from implicit aspects. We used a categorization task, in which the semantic relevance was previously defined based on the congruency between a kept in mind category (abstract knowledge), and the lexical semantic information presented. Our results show that during the detection of the relevant lexical information, phase synchronization of neuronal oscillations selectively increases in delta and theta frequency bands during the interval of semantic analysis. These increments occurred irrespective of the semantic category maintained in memory, had a temporal profile specific for each subject, and were mainly induced, as they had no effect on the evoked mean global field power. Also, recruitment of an increased number of pairs of electrodes was a robust observation during the detection of semantic contingent words. These results are consistent with the notion that the detection of relevant lexical information based on a particular semantic rule, could be mediated by increasing the global phase synchronization of neuronal oscillations, which may contribute to the recruitment of an extended number of cortical regions.

## Introduction

Executive functions have a critical role in supporting language. It has been proposed that these functions are important for language appearance during evolution (Aboitiz and García, 1997; Aboitiz, 2012), for language acquisition (Baddeley, 1992), and for its normal use (Gibson, 1998; Caplan and Waters, 1999), allowing the coordination of sensory and semantic processes across time and accommodating moment-by-moment shifts in goals and strategies (Binder et al., 1997; Smith et al., 1998). During the analysis/creation of the discourse, a critical role of the executive functions relates to the coordination between two different levels of semantic knowledge. One is an abstract knowledge deriving from the combination of words' content across time (propositional semantics), and the other is the low-level knowledge deriving from the incoming lexical material. Roughly, from the combinatorial operations that assemble the basic components (lexical semantic units) into larger structures – a process named “unification” (Hagoort, 2005) – additional information is created and maintained that, in turn, is used to monitor new lexical material. This more abstract level of knowledge, or propositional semantics (Givón, 1995), generates rules that dynamically set the relevance for processed words (Sperber and Wilson, 1987, 1995, 2004), based on its own semantic information. In the definition of these rules, this abstract semantic level requires to be constantly updated with the contingent semantic information detected, in order to adapt to, and to generate, varying propositional information along the discourse (Gernsbacher, 1991, 1995). Thus, it becomes fundamental to understand how the contingent lexical information is detected and incorporated, when it is based on a more abstract semantic representation.

The notion that the referential content created during the discourse sets the specific weights to the new lexical information is contained in the psycholinguistic concept of semantic context. Context is typically viewed as information that either enhances or instantiates a context-independent core representation or as a correlated constraint in which information from higher-level representation can, in principle, inform linguistic processing to lower levels of representation (Tanenhaus and Brown-Schmidt, 2008). An important question derived from this notion is whether and how, semantic context affects lexical processing (Tanenhaus and Brown-Schmidt, 2008). From a neurobiological perspective, this debate involves questions about the architecture of the processing system and the flow of information between different types of representation. Because of the increasingly close alignment between language research and systems neuroscience (Poeppel et al., 2012), modern biological models of language processing incorporate for both, the architecture of the processing system and the flow of information, the concept of “functional-connectivity” in the brain (Pulvermüller, 2002; Pulvermüller et al., 2009). A known fact derived from systems neurobiology is that even for single encoded elements, the information in the brain is widely distributed, and representations may emerge by virtue of the generation of functional communications between the separated coding regions. In fact, the visualized image of a cup, for example, is totally decomposed in its elementary components before it arrives to the visual cortex, and its characteristics (borders orientation, color, texture, location, etc) are parsed in different regions of the cortex. Because of that, the mental recreation of the observed cup requires its cerebral reconstruction, a process that is apparently mediated by functionally coupling the different cortical regions decoding their characteristics, as a unique and specific temporo-spatial activity pattern (Varela et al., 2001). In the context of semantic analysis, it has been proposed that the same process takes place in the brain, but in this case to create an internal memory representation that depends on the life experience, and that in the case of speech is evoked by the acoustic material listened to. This memory representation would cluster uni- and multi-sensory information traces that have been ontogenically associated with the concept itself. This notion also extends to the large-scale functional communication between regions maintaining a high-level abstract representation and those generating a lower-level lexical interpretation, thus integrating the word meaning into an unfolding discourse representation.

In this framework, the central question addressed here is what physiological mechanisms account for an enhancement of the lexical information defined as relevant based on specific rules deriving from more abstract semantic representations. We hypothesized that neuronal oscillations play a pivotal role in the identification and incorporation of relevant lexical information. Our overall neurobiological proposal is based on previous findings showing that: (i) there are anatomical cortical areas (particularly the dorsolateral prefontal cortex, DLPFC) supporting the function of on-line monitoring of the incoming information based on an abstract knowledge (function also defined as “using the rules of the game;” Miller, 2000; Miller and Cohen, 2001); function that has been extended to the language domain by control-based theories, which include the role of the left inferior frontal gyrus (including Broca's region) in the selection of competing semantic information (Thompson-Schill et al., 1997; Hagoort, 2005; Novick et al., 2005; January et al., 2009); (ii) the existence of a neural substrate in the prefrontal cortex supporting the function of defining the relevance for incoming information (Rainer et al., 1998) in general cognitive domains, and (iii) that the oscillatory activity in the brain is an integral part of the semantic analysis (Hagoort et al., 2004; Bastiaansen et al., 2005). We propose that the identification and enhancement of contingent lexical information based on abstract knowledge is mediated by a transitory strengthening of the communication between frontal areas (monitoring and defining the relevance of semantic information) and posterior areas (decoding lexical semantics). Because oscillations enable the cortex with the necessary gating mechanisms for the generation and increase of information fluxes, by means of the establishment of phase relations (Singer, 1999; Varela et al., 2001; Buzsáki and Draguhn, 2004; Fries, 2005; Lakatos et al., 2005), a transient increase in the phase synchronization of the oscillatory activity could be critically involved in the recruitment and functional coupling of cortical areas for the internalization of the new information.

In the present work, despite being ultimately interested in the semantic processing at the natural discursive level, we restricted our experimental approach to a controlled task in which the detection of relevance was based on inferences derived from explicit information, thus controlling for ambiguities derived from implicit aspects of decoding. Because in the context of the discourse, the relevance for the lexical semantic information can be constructed based on different aspects of the propositional semantic created, which have also subjective dependencies, the use of a naturalistic situation can hinder the interpretation of the supposed relation between an abstract semantic rule and the target lexical material. We therefore used a categorization task in which the semantic relevance for the lexical information presented was previously defined based on the congruency between a kept in mind category (abstract knowledge) and the semantic content of words aurally presented to the subject. The rationale of our experimental task was that at the base of the paradigm is the construction of abstract semantic knowledge (which in this case we recreated by the maintenance of a particular semantic category), and a specific rule that defines lexical semantic relevance (in our task, under the instruction to look for the semantic congruency). Words of different semantic categories had to be semantically analyzed but differentially responded to, thus equating the cognitive requirements of analysis and the need of motor response for all categories, which allowed us to explore the specific influence that the abstract knowledge maintained in mind has on the detection of relevant semantic information.

## Materials and methods

### Participants

Sixteen right-handed native Spanish speakers (10 females), age 20–33 years, participated in the study, signing a written consent. The subjects had no history of neurological disorders, did not abuse alcohol or drugs, had normal hearing, and shared similar socioeconomic and educational levels. All subjects finalized the task and none of them reported discomfort.

### Stimuli and task

Two independent but complementary studies were separately applied to different subgroups of subjects (*n* = 8 each group). The first study was aimed to evaluate the effect that keeping in mind a semantic category has on the functional coupling (phase synchronization) during the analysis and detection of contingent lexical material, compared with the non-relevant (competing) lexical information. The second was a follow-up study aiming to control for specific effects of different kept in mind categories. Thus, the goal of the second study was to ascribe results of phase synchronization modulation, to the phenomenon of monitoring lexical information based on an abstract semantic representation, independent of specific semantic categories. Both studies consisted of a variation of the classical Lexical Decision Task (LDT), where Spanish words pertaining to three different semantic categories (i.e., animals, man-made objects, and abstract nouns), and pseudo-words, were presented binaurally. In the first study, subjects were informed about the inclusion of different semantic categories and pseudo-words, but only one was explicitly named (animals), and instructed to be “kept in mind” during the whole session. The behavioral task was to press one of two possible buttons, contingent to whether the heard word belonged or did not belong to the instructed category. This forced subjects to semantically analyze each word irrespective of its content or category, and also equated the motor response requirements for each word. For the second study, stimuli were presented in a block design (three blocks). Each block consisted of the presentation of words belonging to two different semantic categories and pure tones (i.e., three different categories). Categories included in each block were, man-made objects and abstract nouns; animals and pseudo-words; pseudo-words and man-made objects, respectively. Kept in mind categories for each block were, man-made objects, animals, and pseudo-words, respectively. Note that the third block contained words belonging to the first and second categories. The rationale behind this approach assumes that the first and second blocks examine the consequence of a first exposure to those stimuli, whereas the third block examines the consequence of a repetitive exposure, in which, in addition, we rotated the cognitive requirements for each category. This manipulation allowed us to evaluate the weight that the cognitive process under study has, compared to semantic categories and exposition, in the results obtained. The use of the categories “animals” and “man-made objects” was used to contrast living versus non-living categories. Abstract nouns were included to evaluate differences in the processing of non-visual conceptual information. Pseudo-words and pure tones were utilized to contrast processing related to verbal non-semantic information in the case of the first one, and auditory non-verbal information for the second. All of these selections were designed to evaluate specific topographic differences between different types of semantic and non-semantic information in the context of oscillatory activity, which become the objective for a complementary study.

Commercial computer programs controlled all aspects of the tasks (Stim, NeuroScan Inc., USA, for the first study; Experiment Builder, SR Research Ltd., Mississauga, Canada, for the second study). Verbal stimuli consisted of Spanish disyllabic (consonant-vowel-consonant-vowel) words, and equally structured pseudo-words, which contained syllables existing in the used words. The frequency of use of the words included in the stimulus set was evaluated in an independent sample of 10 subjects with equivalent educational level, using an analog scale from 1 to 10 points applied to a pool of 40 words for each category. A group of 10 words were selected for each category that were rated over seven points. Categories were defined as: man-made objects (e.g., mesa), animals (e.g., gato), abstract nouns (e.g., nota), pseudo-words (e.g., mepo) and pure tones. Each category contained 10 stimuli, which were repeated six times during the task (60 stimuli for each category). Verbal stimuli were digitized using a female voice (A/D rate = 22050 Hz, 16 bits, average duration around 500 ms); pure tones were digitally constructed (frequencies between 280 and 640 Hz; duration 500 ms) and amplitude modulated applying a symmetric envelope of 250 ms to simulate the amplitude modulation proper of syllables (Audacity® 2.0.0; free digital audio editor). The amplitude of all stimuli was normalized to equate their magnitude and presented at 80 dB SPL. Sequences of words or tones were pseudo-randomly presented (SOA: 1.7–2.3 s), preventing consecutive intra-category appearance to eliminate semantic priming effects. Stimuli were delivered through commercial earphones (NeuroScan Inc., Neuromedical Supplies, USA) to subjects seated in a comfortable chair with eyes closed, in a sound-attenuated and electrically shielded room.

### Recordings

For the first study, an 80-channel montage and two 40-channel amplifiers (Quik-Cap and NuAmps, NeuroScan Inc., Neuromedical Supplies) were used for data collection. Vertical and horizontal electro oculograms were recorded. Cz served as reference for acquisition, and Afz as ground. EEG was collected continuously (A/D rate = 1000 Hz, 32 bits precision, filters = DC-100 Hz). We used Scan 4.3 software (Neurosoft Inc., USA) for initial data processing. Offline filter settings were: high pass over 0.1 Hz (Butterworth, zero phase-shift filter, 24 dB/Oct). For the second study, a 32 + 8-active channel montage and a 32 + 8-channel amplifier (BioSemi ActiveTwo System; http://www.biosemi.com) with a CMS-DRL reference system was utilized. EEG was collected continuously (A/D rate = 2048 Hz, 24 bits precision, filters = DC-1024 Hz). Channels not reaching impedance below 5 Kohms were eliminated from the analysis. Epochs were extracted in the interval from −1000 ms to 2000 ms around stimuli. Baseline activity was defined as the 500 ms preceding the stimulus presentation. Epochs containing signals in frontal channels exceeding ±100 μV were eliminated, as well as signals containing great eye movements, electromyographic, or other visually identified artifacts.

### Phase-synchrony analysis

All subsequent analyses were carried out using MATLAB. Data was imported by means of EEGLAB toolbox package routines (Delorme and Makeig, 2004) using averaged-mastoids signal as reference. To eliminate remaining blinks, ocular, and electromyo-graphically generated noise, the EEG signals concatenated across all trials were decomposed using Independent Component Analysis (ICA). Artifact components were visually identified, based on time series and their topographic distribution. These components were extracted and the signals re-synthesized. Then, the EEG signals of each subject were re-referenced to the average of all EEG channels. To compute phase-locking values (PLV, Lachaux et al., 1999), phase was obtained from the Fourier coefficients calculated by means of a short-time Fourier transform (STFT). Time-frequency decomposition was computed on the EEG epochs tapered by a sliding Hamming window, using different window lengths for specific frequency ranges (1–4 Hz: 1024 ms; 4–8 Hz: 512 ms; 10–20 Hz: 256 ms; 20–40 Hz: 128 ms) applied in steps of 50 ms. To avoid edge effects, the first and last 200 points of the time series were merged to the initial and final epochs endings points, respectively, as a flipped, reflected copy, and the regions of interest were finally defined 500 points away from the original extremes. For each computed coefficient, the phase (ϕ) was obtained as the arctan of the complex number. With this angle, a complex vector of unitary magnitude was constructed. This way, a complex valued phase vector was obtained as function of electrode, time, frequency, and trial number. Phase differences between all pairs of selected electrodes were then calculated for each frequency, time and trial, and then averaged across trials. By the modulus of this complex average value, we obtained a magnitude of phase difference, which could vary between 0 (random phase relation) and 1 (constant phase relation). That is, being Φ_*i*_ (*f, t, k*) the phase value of electrode *i*, at frequency *f*, time *t*, and trial *k*, and Φ_*j*_ (*f, t, k*) the phase value of electrode *j*, in the same frequency, time and trial, the phase difference was computed as $\Phi_{ij}(f,t)=\frac{1}{N}*\left|\sum_{K=1}^{N}\Phi_{i}-\Phi_{j}\right|$. In order to eliminate volume conduction effects, phase differences of 0 or 180° were discarded previous to compute the complex average vector (Melloni et al., 2007). Values of phase differences were then transformed to *Z*-Scores normalizing at each frequency by the respective values obtained during their baseline interval, as Pn_(*f*)_ = (P_(*f*)_ − μ_(*f*)_)/σ_(*f*)_; where P_(*f*)_ is the phase difference value obtained at frequency *f* and each time point across trial, and μ_*f*_ and σ_*f*_ are the mean and standard deviation of the phase difference values during the baseline at the same frequency.

Statistically significant phase-synchrony (PLV) was calculated by comparing the *Z*-Scores of phase differences obtained for original signals, with phase differences equally computed in epochs of shuffled signals generated by the following procedure: the signal of each trial was subdivided into windows of the same size as the windows used in the time-frequency decomposition. These windows were randomly redistributed, resulting in trials with random phase values in each electrode. Finally, a Wilcoxon test between phase differences of recorded signals and 200 shuffled signals, was then used to establish a statistical significance of the phase differences obtained during the task (Lachaux et al., 1999).

Because the topographic distribution of the scalp activity produced is specific for each semantic category (Murphy et al., 2011), the comparison of phase coupling values between conditions, based on their differences of scalp distributions, is not well suited for the present study (i.e., differences between conditions could reflect inherent differences in the activity patterns generated for the lexical material analyzed, and not the cognitive effect under study). To evaluate statistical differences in the global phase synchronization between conditions, we compared the average phase synchronization values obtained at different time-frequency regions of interest (across all electrode pairs that reached a significant PLV in those windows of interest). A Kruskal–Wallis test was applied to those time-frequency regions, with Tukey's multiple comparisons – when K–W Chi-square statistic was significant, to compare phase synchronization between conditions.

To compare the effect of the experimental manipulation between the first and second studies, as they had different subjects populations and design, and to evaluate a possible interaction between tasks, we applied a Two-way analysis of variance (ANOVA) to the phase synchronization results with the factors category (attended versus not attended) and group (1 versus 2).

### Global field power

Global field power (GFP) was computed for each category to rule out the possibility that differences in the global phase synchronization values between categories were reflecting differences in the strength of event related evoked activity. To this end, signals were filtered between 0.01 and 20 Hz and the average event related potentials (ERP) for each subject was first computed. GFP was computed as standard deviation of the momentary potential values across electrodes (Lehmann and Skrandies, 1980) for each subject, and then averaged across all of them.

## Results

All subjects completed both tasks, while none of them reported discomfort. For the first study, the mean number of rejected epochs for each category was 17 ± 6.8, 20 ± 6.9, 17 ± 5.7, and 19 ± 6.8, respectively and the average of channels used was 58 ± 5.8 for all categories. For the second study, no epochs and channels were rejected. Because the conjunction of brain loci involved in semantic analysis is specific for each semantic category, and even for single concepts (Tranel et al., 1997b; Murphy et al., 2011), we did not select a common group of electrodes to compare between categories (see Materials and Methods). In addition, there is no published data regarding which groups of electrodes would become synchronized in our task. Moreover, modulations of phase synchronization can result as a consequence of the recruitment of new brain areas during the task, with a corresponding increase and/or reduction of phase synchronization values between different pairs of electrodes. This assumption advises against the application of any *a priori* criterion to select a region of interest for comparing between categories. Therefore, we evaluated the distributions of variables across all valid pairs of electrodes that reached statistically significant phase synchronizations and used the averages across electrodes to make all between-conditions comparisons. For both studies, categories were numbered as 1 (man-made objects); 2 (animals); 3 (abstract nouns), and 4 (pseudo-words).

### First study

#### Phase synchronization during semantic analysis

**Figure 1:**
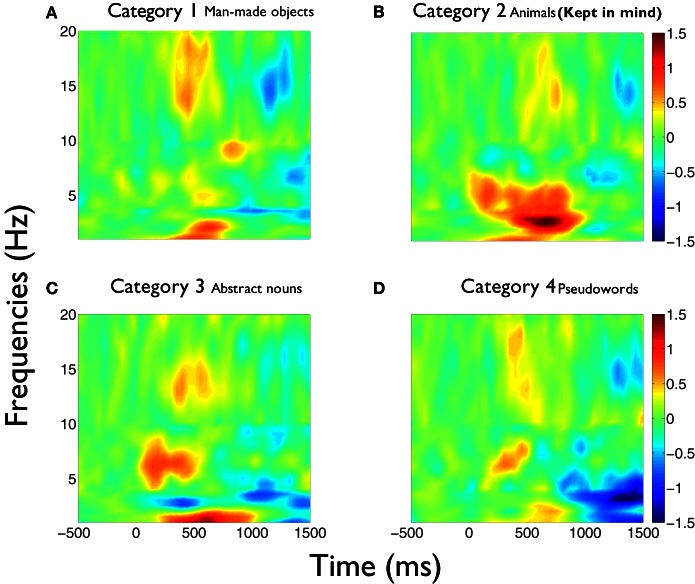
**Phase synchronization increments during semantic analysis are larger for the kept in mind category in delta and theta frequency bands.** Average time-frequency charts of the phase synchronization changes (*Z*-scores) obtained during the task, for each category [**(A)** category 1, i.e., man-made objects;**(B)** category 2, i.e., animals; **(C)** category 3, i.e., abstract nouns; **(D)** category 4, i.e., pseudo-words]. **(B)** Corresponds to the kept in mind semantic category. Each chart represents the average of the *Z*-scores values across all statistically valid pairs of electrodes and subjects. Major changes are observed in delta, theta, and beta frequency bands. The increments produced in delta and theta bands in the category 2 **(B)** are significantly larger compared to the other categories in all time intervals and frequency ranges of interest (see text for detailed explanation).

We first outline the results of the first study. Figure 1 shows the time-frequency charts of the average phase synchronization changes produced during the task, for each category between 1 and 20 Hz. These graphs show that the principal increment in phase synchronization after word presentations occurs, for all categories, in the delta and theta frequency bands. Also, an increment in phase synchronization at beta frequency band is apparent for all categories, which is localized in time at around 500 ms. In this case, the category number 2 (animals) corresponded to the kept in mind category. Many differential features become evident in the global phase synchronization pattern of category 2 compared to the other ones. First, whereas in categories 1, 3, and 4, delta increments concentrate in the frequency range between 1 and 2.5 Hz in the time interval between 250 and 800 ms, in the category 2, delta increment spanned continuously the frequency range between 1 and 4 Hz, and became continuous with the increment in theta band. Moreover, whilst in categories 1, 3, and 4 there is a frequency range of desynchronization between 2.5 and 4 Hz, in category 2 this is the frequency range where increments of synchrony become prominent and more prolonged. Second, in all categories there is an early increase of synchrony in theta band in the range of 5–7 Hz between 150 and 250 ms. While in all categories there is a second increment at theta band around 500 ms, in category 2 this increment is continuous between 300 and 850 ms.

**Figure 2:**
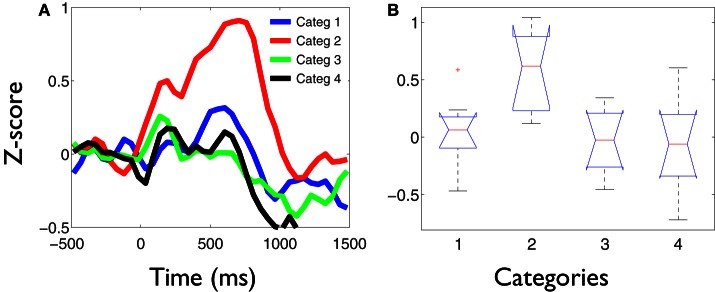
**Delta-theta phase synchronization increments are significantly larger for the kept in mind category. (A)** Temporal profile of the average phase synchronization changes during the task, in the frequency range between 1 and 6 Hz for each category. Each signal represents the average of the *Z*-score values across all valid pairs of electrodes and subject, during the task. At all temporal-frequency windows, defined as regions of interest within these bands, the average phase synchronization increments for the kept in mind category (2) were significantly larger compared to the other categories (Kruskal–Wallis test, P < 0.01; Tukey's multiple comparisons). **(B)** Example box and whisker plots of the phase synchronization values (*Z*-scores) obtained between 1 and 6 Hz in the time interval between 250 and 800 ms poststimulus. On each box, the central mark depicts the median, the edges of the box represents the 25th and 75th percentiles, the whiskers extend to the most extreme data points not considered outliers, and outliers are plotted individually. Notches draw comparison intervals.

**Figure 3:**
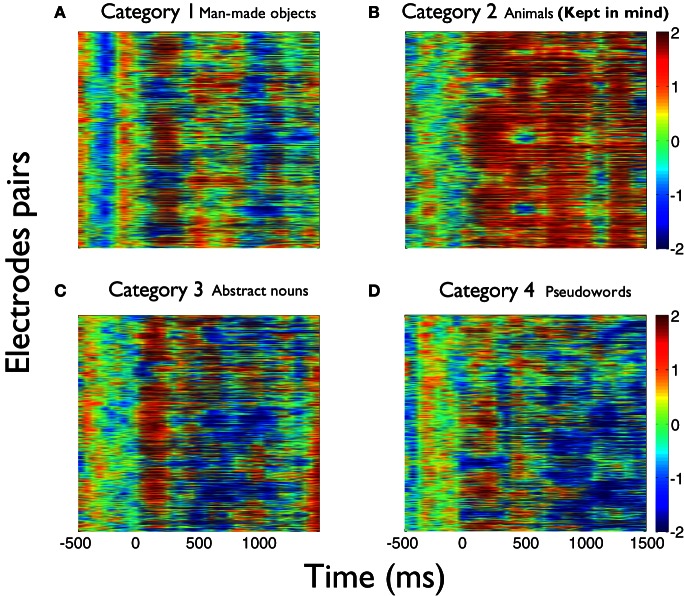
**Detecting relevant semantic information selectively synchronizes new electrode pairs during the task.** Example plots of the phase synchronization changes at 4–6 Hz occurred between all valid pairs of electrodes for each category during the task, in one subject. Categories **(A–D)** are ordered as in Figure 1. **(B)** corresponds to the kept in mind category. Note that there are specific time windows where electrodes become synchronized across the task in each category. Category 2 **(B)** is characterized by a marked recruitment of new electrode pairs that increase the global phase synchronization, which could be associated to new areas becoming active during the analysis and detection of words pertaining to the relevant category. As in the average across all subjects, global phase synchronization increments not only become larger, but also more prolonged for the kept in mind semantic category **(B)** than for the others **(A,C,D)**.

The values of the phase synchronization changes across the session (*Z*-scores) were normally distributed for all categories in the time intervals of interest. For the first study, mean and maximal *Z*-score values in the range of 1–3 Hz for each category, in the interval between 250 and 800 ms, were: 0.44 and 11.14; 0.68 and 16.27; 0.37 and 10.22; 0.27 and 15.13, respectively. In the interval between 500 and 800 ms, mean and maximal *Z*-score values in the range of 3–4 Hz for each category were: −0.06 and 12.16; 1.13 and 12.11; −0.28 and 10.26; −0.04 and 11.74, respectively. Finally, in the interval between 350 and 800 ms, mean and maximal *Z*-score values in the range of 4–5.5 Hz for each category were: −0.18 and 24.6; 0.67 and 21.67; −0.12 and 25.27; −0.15 and 25.93, respectively. These distributions show that the task is associated with strong modifications of the functional connectivity observed between electrodes at baseline times. This modulation is manifested through strong synchronizations and desynchro-nizations at different electrode pairs, latencies, and frequency bands. Overall, these synchronization modulations are best characterized as a net global increment of the inter-electrode phase synchronization for the kept in mind category, at all time intervals and frequency ranges examined (Kruskal–Wallis test, χ^2^ = 11.82; P < 0.01; Tukey's multiple comparisons; for the average signal between 100 and 800 ms poststimulus) (Figure 2). Figure 3 shows an example of the temporal profile of phase synchronization changes that occurred between all valid pairs of electrodes during the task, for each category, in one subject. The figure displays the phase synchronization modulation that occurred in the frequency range between 4 and 6 Hz, and shows dynamic inter-electrode phase synchronization changes having an ordered temporal profile, even in the categories displaying more discrete synchrony changes. This result indicates that synchronizations and desyn-chronizations between electrodes occurred as ordered phenomena at specific latencies during the task. Category 2 showed the largest and prolonged increments.

**Figure 4:**
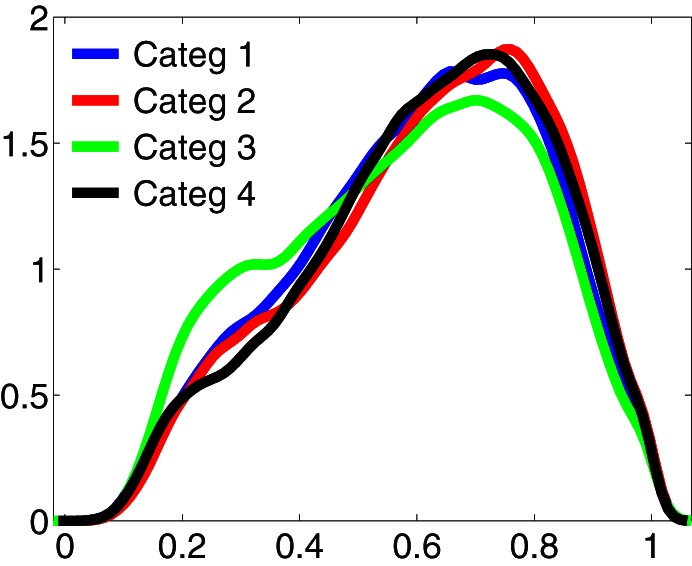
**Stronger phase synchronization for the kept in mind category is not associated to a volume conduction phenomenon.** Probability density functions (PDF) of the non-normalized phase differences between electrodes, obtained in the frequency range between 2 and 4 Hz, in the interval between 250 and 800 ms for each category. This time-frequency region is where the kept in mind category (2) manifested stronger differences with the other categories. Distributions show the existence of a similar amount of phase differences with value closer to 1 between categories. This result was replicated in each time-frequency window of interest across the task, excluding the possibility that the stronger inter-electrodes phase synchronization manifested by category 2 would be the result of a volume conduction effect.

A parsimonious explanation for the greater global phase synchronization manifested by category 2, is that these increments were the product of volume conduction of a focal phenomenon. In order to address this possibility, phase differences of 0 or 180 degrees were discarded prior to computing the average phase synchronization differences between electrodes (see Materials and Methods). Also, if this was the case, it would be expected that in this category, an important proportion of the phase differences obtained between all electrodes had a value closer to 1, when compared to the other categories. To assess this possibility, we compared the distributions of the non-normalized phase difference values of all valid pairs of electrodes between categories for each time interval and frequency range of interest. Figure 4 shows the parametric, kernel-smoothed probability density functions (PDF) of phase differences, computed for each category in the time interval between 250 and 800 ms in the frequency range between 2 and 4 Hz. This figure shows that all distributions contain a similar amount of phase differences with values close to 1, and strongly suggest that the increase of the phase synchronization displayed by category 2 during the task does not occur as consequence of volume conduction effects of a local phenomenon. This was also the case for all time intervals and frequency ranges compared between different categories.

These phase synchronization distributions also suggest that the overall increase of phase synchronization for the kept in mind category (*Z*-scores compared to baseline) does not occur principally as a result of a group of electrodes becoming particularly synchronized, but probably through a recruitment and synchronization of new areas involved in the processing of this category. This is because a greater synchronization of a similar number of electrodes for the kept in mind category, would be manifested as a change in the distribution of phase differences for that category, when compared to the others. While the distribution of phase differences of this category tends to have the greatest values (Figure 4), this is insufficient to explain the greater increment in the average phase synchronization observed for this category (Figure 2). This is also illustrated in Figure 3, where it results evident that in the case of the kept in mind category a greater number of pairs of electrodes become synchronized, compared to the others categories.

#### Evoked activity and global field power

**Figure 5:**
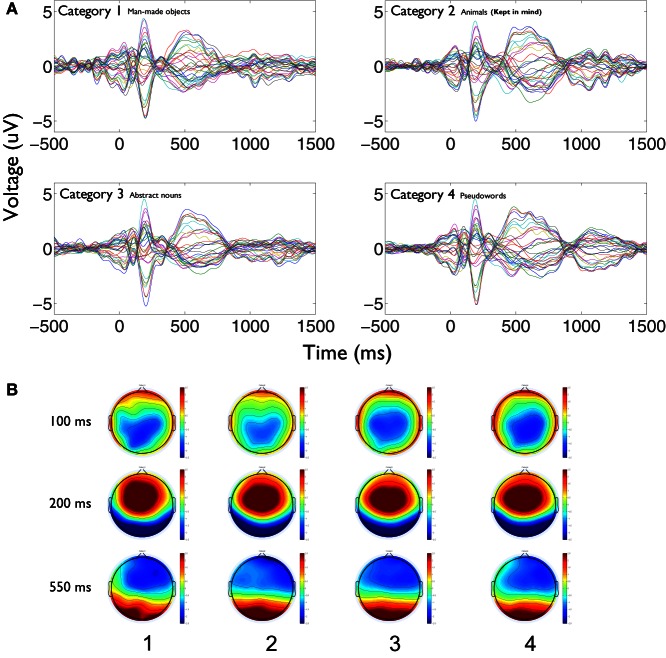
**Stronger phase synchronization for the kept in mind category is not related to the appearance of event related evoked activity. (A)** Event related potentials are displayed as overlaid traces of recording electrodes between −500 and 1500 ms around stimulus presentation for each category. Epochs time series were filtered between 0.01 and 20 Hz. Kept in mind category (above right) does not shows specific components at any latency that could relate to the global increase of phase synchronization profile found for that category compared to the others, not attended ones. **(B)** The spatial distributions of the evoked activity at different latencies (100, 200, and 550 ms poststimulus) is displayed for each category (number 2 is the kept in mind). As time series, spatial distributions of evoked activity are quite similar between all conditions, not showing any marked difference for the attended one.

**Figure 6:**
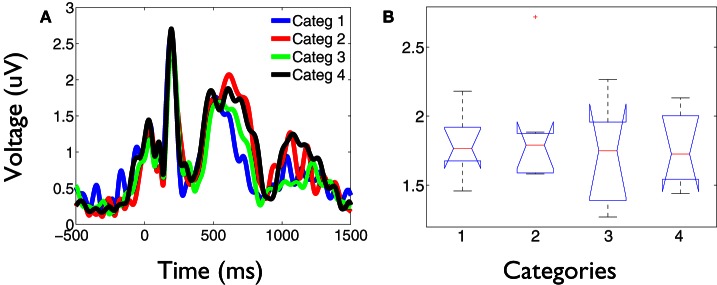
**Stronger phase synchronization for the kept in mind category is not associated to an increase of the time-locked global field power. (A)** GFP obtained for each category during the task. The signals represent the average across all subjects. Despite the intrusive alpha activity, it can be observed that category 2 is not associated to a greater evoked activity at any latency during the task. **(B)** Example box and whisker plots of the average activity across subjects in the time interval between 500 and 600 ms poststimulus, when prominent differences were found in the profile of global phase synchronization between conditions. No significant differences were obtained between category 2 and the other categories at any latency across the task.

It could also be argued that the inter-electrodes phase synchronization increment displayed at low frequencies, in the kept in mind category, were reflecting the appearance of event related evoked components associated to target detection or other types of time-locked cognitive phenomena. Whereas this possibility is unlikely considering that the modulation patterns of phase synchronization found were induced rather than evoked, we addressed this issue by computing the GFP for each category, and measured statistical differences between conditions at each poststimulus latency. Because GFP is a parametric assessment of map strength, it gives a good measure to evaluate differences in the appearance of global evoked activities between conditions. A similar profile of the GFP across time was observed for all categories. Figure 5 displays first the ERPs obtained for each category, attended and not attended, as overlaid traces of the recording electrodes (Figure 5A) and the topographic distributions of scalp potentials at different poststimulus latencies (Figure 5B). Time series and spatial distributions of ERPs are quite similar between conditions, and in the case of the attended one, particular evoked activities are not evidenced at any latency that could explain the strong differences found in the global phase synchronization profile between the attended and not attended conditions (see Figure 2A). We did not apply permutations analysis to evaluate specific spatial differences between conditions because, as previously mentioned, differences could be inherent to the semantic material analyzed that recruit different patterns of cortical areas, and not to the cognitive phenomenon under study. The point that we want to stress here is that there is not a global increase in the strength of the evoked activity, at any latency, as observed in the phase synchronizations patterns between conditions. Figure 6 shows the GFP obtained for each category during the task. Some intrusion of alpha oscillations in the global field activity is observed, which was found in all subjects that performed the task, with eyes closed in a light attenuated room. For all latencies after stimulus presentation, we found no statistical differences between the GFP for category 2, the attended one, compared to the other categories (Kruskal–Wallis test, P > 0.05; Tukey's multiple comparisons). These results demonstrate that the global phase synchronization differences observed between this and the other categories can not be attributed to the generation of event related evoked activity during the task.

### Second study

#### Phase synchronization

**Figure 7:**
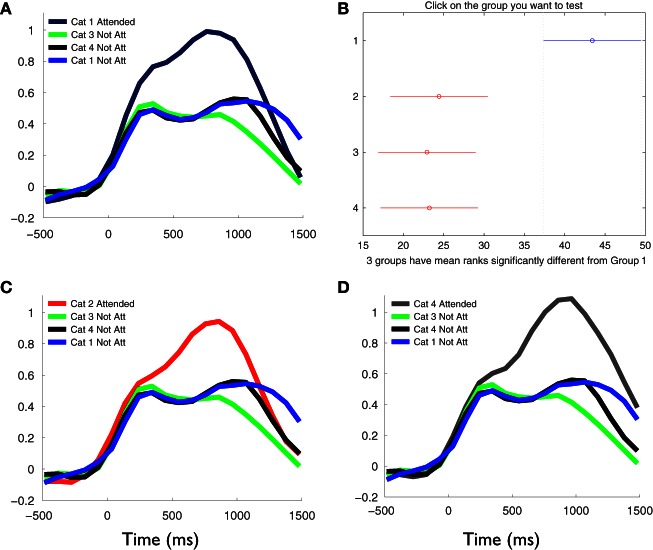
**Stronger phase synchronizations are dependent of the cognitive task and occur independently of the semantic material to be kept in mind.** Temporal profile and statistical difference of the average phase synchronization changes during the second phase of the task, in the frequency range between 1 and 6 Hz for each category. Each signal represents the average of the *Z*-score values across all valid pairs of electrodes and subject, during the task. **(A)**, **(C)**, and **(D)** show the comparison between the kept in mind category at each block of the task, respectively, and the same and other categories when not attended in all blocks. Note that kept in mind categories produced larger global phase synchronization compared to the non-attended categories [Kruskal–Wallis test, P < 0.05; Tukey's multiple comparisons; **(B)**]. This phenomenon occurred independently of categories. Moreover, the intra-category comparison of the phase synchronization magnitudes produced when it was kept in mind and when neglected, showed that the increments of synchronization are due to the cognitive process of extracting semantic relevance for the lexical information and not depend on the semantic material *per se*.

In the second study, we addressed the possibility of our results being specific for the animals category or whether previous results would generalize to any semantic category, thus attributing the increment of phase synchronization to a general cognitive process of detecting relevant information for a kept in mind conceptual representation. In this case we constructed blocks of stimuli presentations, varying now the category to be kept in mind (see Materials and Methods). In order to compare the cognitive effect between conditions, we contrasted the results of each category when it was kept in mind, to others and the same category, when they were not relevant to the task. Time-frequency plots of the phase synchronization changes for all categories showed similar profiles to that observed in the first task, where major increments of phase synchronization were again evident at delta and theta frequency ranges (data not shown). We therefore averaged *Z*-score values of phase synchronization between 1 and 6 Hz, in order to evaluate the changes between conditions. Figure 7 shows the temporal profile of these changes. As in the first experiment, in the categories that were kept in mind, the detection of the relevant lexical information produced a greater global phase synchronization compared to the non-attended categories (Kruskal–Wallis test, χ^2^ = 6.95; P < 0.01; Tukey's multiple comparisons; for the average signal between 200 and 800 ms poststimulus. Figure 7A). This phenomenon occurred irrespective of the kept in mind category. Furthermore, when we contrasted the phase synchronization changes that occurred in the same category when it was kept in mind and when it was not, significant differences occurred in the pattern of global synchronization (Figures 7A,D), showing that the increased synchronization may be attributed to the cognitive process of extracting semantic relevance for the lexical information and not to basic context free lexical processing. Interestingly, this was also observed when the kept in mind category corresponded to pseudo-words, demonstrating that our results generalize and ultimately reflect, a complex cognitive process of detecting relevant incoming information based on an abstract rule. The comparison of the phase synchronization results between both tasks (first and second studies) by means of a Two-way ANOVA with factors category (attended versus not attended) and group (1 versus 2), showed a significant effect of the category (*F* = 8.15; *P* < 0.001) and group (*F* = 18.16; P < 0.001) but no interaction between both factors (*F* = 0.37; P = 0.78). These results show that the difference of global phase synchronization between categories, i.e., the effect of attend versus not attend to categories, is maintained and independent of both studies applied.

#### Topographic maps of phase synchronization changes

**Figure 8:**
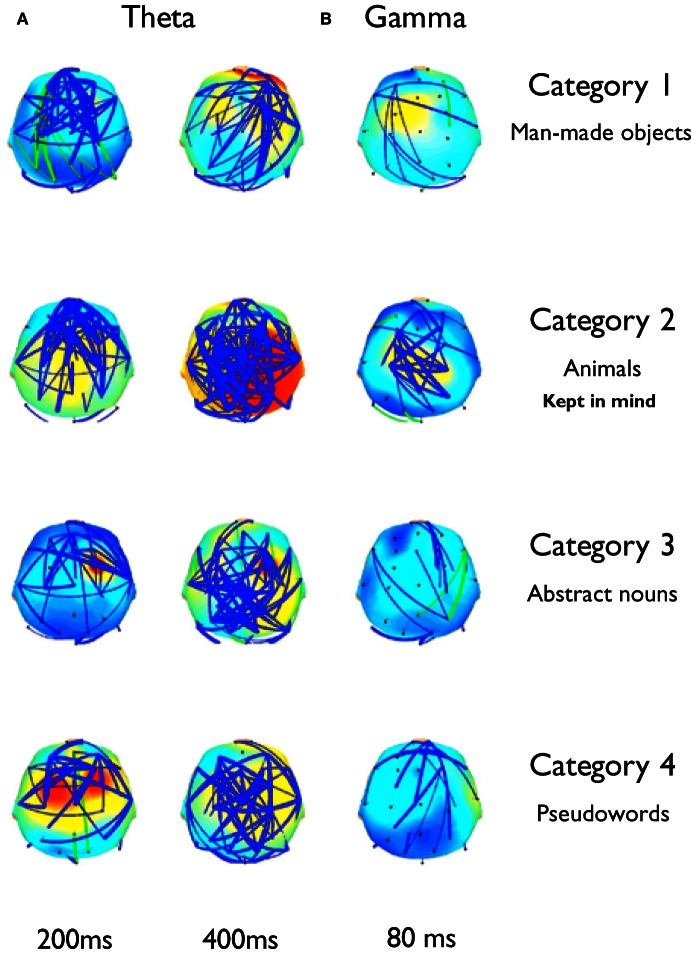
**Significant phase synchronization established between electrodes at different latencies in theta and gamma bands.** Topography maps show the average significant phase synchronization established across all subjects. Whereas each subject displayed a specific topography pattern for each category, the maps show a gross average view of the inter-electrodes phase synchronization at different latencies in theta and gamma bands. **(A)** At 200 ms, phase synchronizations in theta are generated principally at frontal locations, with category 2 showing an extended pattern to more posterior areas. At 400 ms this pattern incorporates an increased number of posterior regions in all categories, and category 2 recruits an increased number of electrode pairs compared to the other ones. **(B)** At 80 ms, there was a transient increase of phase synchronization of the gamma activity around 40 Hz for all categories. While this increase was not significantly greater for category 2 compared to the others, this could be related to the fact that these increments are topographically restricted than in the other frequency bands, and statistics are applied over the whole set of electrodes.

Our results show that during monitoring of the semantic content of words while keeping in mind abstract semantic knowledge, increments of phase synchronization were significantly higher during the analysis of words whose content was contingent to the task. These increments were prominent at delta and theta frequency bands, and despite the fact that they displayed a specific temporo-spatial pattern for each subject, global patterns can be inferred from these changes. Whereas theta increments appear in the form of two major time epochs up to 500 ms in the non-attended categories, the second increase prolongs at least until 850 ms for the kept in mind category. We generated topographic maps of these theta increments to obtain an average image of the spatial pattern of phase relations established for each category (Figure 8A) These maps show that the first increment at 200 ms involves principally frontal electrodes. However, for the kept in mind category these phase relations show an extended pattern involving more posterior regions (Figure 8A). At the second latency (400 ms), theta increments clearly involve more posterior regions in all categories, but again, the phase relations established in the attended category involved an increased number of electrodes compared to the other categories. We plotted maps of synchronization at gamma frequency band (around 40 Hz) because we observed an early increase around 80 ms for all categories (Figure 8B). However this increase was not significantly higher for attended categories. This may be explained in part because the statistical tests applied considered the complete set of electrodes, and changes in this frequency band were more locally restricted. For all categories, there was a discrete increase of phase relations at this frequency band, but for the attended category, this increase concentrates at central locations, where the activity of early auditory regions tends to be projected (Figure 8B). Delta synchronizations, as those of theta band, also showed a significantly higher and more prolonged temporal pattern for the attended categories. This phase synchronization increment spanned the period of the theta increments, which suggests the possibility that a functional relation between these frequency ranges was established. In all categories, attended and unattended, we observed a temporally localized increase of the phase synchronization in the beta band at around 500 ms (Figure 1). We hypothesize that, because of its temporal location, this activity could be related to the preparatory activity associated to motor response. Interestingly, median reaction times were higher for the attended categories (0.71, 0.8, 0.75, and 0.77 seg. respectively for each category, in the first task), which is in association to the more prolonged delta and theta increments, which in turn could be related with a time consuming processing taking place during the analysis of the words pertaining to that categories, like the internalization of phonological and/or semantic information in memory.

## Discussion

It has been widely proposed that the neurophysiological integration of sparse information in the brain is achieved by means of the synchronization of the neuronal activity across local coding regions (Singer, 1999; Varela et al., 2001; Fries, 2005). As previously mentioned, oscillations enable the cortex – by establishing phase relations – with the necessary mechanisms of gating for the generation of information fluxes across brain regions (Singer, 1999; Varela et al., 2001; Buzsáki and Draguhn, 2004; Fries, 2005; Lakatos et al., 2005). In this context, neuronal oscillations, by means of their phase synchronization, would be critically involved in lexical decoding and integration, mediating the binding of the sparse electrical activity representing specific characteristics of the semantic construction.

The main question we addressed in the present study derives from the fact that language is not interpreted as a sequence of equally weighted lexical units, but in the context of a propositional semantic that is constructed as utterances unfold. As complex conceptual information is constructed across time, an interaction is required between different levels of semantic knowledge. Early models of language analysis included the notion that the complex conceptual information created set the relevance (weight) for incoming lexical information, based mainly on the limited working memory capacities (Tanenhaus and Brown-Schmidt, 2008). Because of these limitations, it has been proposed that only specific lexical units are incorporated to update the abstract knowledge, based on the defined relevance. Considering the neurobiological substrate for supporting this function, it has been suggested that frontal areas play a crucial role in the maintenance of an abstract semantic representation, and also of the rules defining the relevance of incoming information, thus allowing the monitoring of lexical information (Thompson-Schill et al., 1997; Hagoort, 2005; Novick et al., 2005; January et al., 2009). The critical element we incorporate in this framework and that we tested in the present study is that phase synchronization of the oscillatory activity participates in the interaction between the different levels of semantic representations. We propose that, whereas the new lexical material is recreated in posterior sensory, motor, and multimodal areas, the matching between this information and the “relevance rules” would be produced by functionally coupling these posterior and frontal regions. When the incoming semantic information is detected as relevant, this functional coupling is strengthened in order to enhance and incorporate it to the conceptual knowledge maintained. Because phase synchronization may allow the strengthening of the functional coupling, we predict that: (i) while the semantic information is being decoded, functional bridges are established with frontal areas to detect the relevance of information, (ii) when the semantic relevance of words is detected, these established phase synchronizations selectively increase, probably recruiting new cortical areas.

As we previously described, despite the fact that our hypothesis concerns the physiological mechanisms accounting for the interaction between different levels of knowledge in the natural language condition, in the present work we decided to reduce the study to a controlled condition. Whereas this clearly does not allow the generalization of the current results to natural language, it gives a first insight about the process to project future studies. In the context of our task note that, whereas we used the knowledge of a category as the more abstract level of semantics, this does not imply that we assume that semantic relevance is based on the knowledge about categories. In fact, semantic contingency could be based more on the pragmatic knowledge that defines what we are talking about (Sperber and Wilson, 1995), which could have at some moment little or nothing to do with specific categories. For example, we could be talking about the care of racehorses, in which case it could be of higher relevance to talk and incorporate the information about a horseshoe (a man-made object) than to talk about a zebra (a very related animal). Also, the hypothesis neither imposes the condition of congruence between the semantic contents to define the relevance of the information. In the case of a psychiatrist trying to catch information about the discourse of a probable schizophrenic patient, the relevant rules will probably be constructed over the base of an existent semantic incongruence. However, we cannot argue that the neural phenomena occurring in this last case would be the same as in congruent conditions, because it could be that different processes take place (Ortega et al., 2008) given that the more natural condition of relevance in the brain is the congruence.

More studies are needed to address specific aspects of the dynamic phase synchronizations generated during the processing of lexical information in the context of propositional analysis. One of these aspects relates to the specific role that different frequency bands play in the process at different latencies. As it has been recently proposed (Giraud and Poeppel, 2012) theta and gamma oscillations can play a crucial role in the extraction of the temporal properties of speech at early stages of processing, allowing an ordered output of linguistic attributes to superior cortical levels for the analyses of more complex characteristics. Because of the frequency of the syllabic units, theta plays a fundamental role in the organization of the information at these stages. Theta is also fundamental for the extraction of semantic information (Hagoort et al., 2004; Bastiaansen et al., 2005). In this context, the activity related to these different levels of processing would be probably intermingled at different temporo-spatial scales, requiring more complex analysis and/or recording tools. Considering our findings, for example, theta could be accounting for the extraction of acoustic elements in rhythmicity with the syllabic structure, as proposed by Giraud and Poeppel (2012), and these “packages” of information can be temporally unified to construct semantic information by means of delta activity. Our results are in agreement with this scenario, because theta synchronizations appear in specific time epochs with a temporal relation with the syllabic structure (Figure 1). Both transient synchronizations can be linked by the delta synchronizations that spanned the period of both theta increments. In the case of the kept in mind category, the more prolonged second theta synchronization can be related to strengthening or a rehearsing of the phonetic information contained in syllables for its association and incorporation with the semantic information in memory. In the same category delta increments are also more prolonged accompanying theta, supporting its unifying role for semantic processing. At the same frequency band in the other categories, there is a net desynchronization phenomenon, which could be accounted for by the inhibition of the incorporation of this semantic information in memory, because of its interfering effect for the task. In any case, more refined analyses will be required in order to account for the complex spatiotemporal dynamics of these synchrony patterns across multiple cortical regions.

Semantic analysis at the lexical level is explained by modern theories in terms of “co-activation of representational nodes” (Small et al., 1995; Pulvermüller, 1999, 2002; McClelland and Rogers, 2003). This notion derives from neuroanatomical (McCarthy and Warrington, 1988; Hillis and Caramazza, 1991; Hart and Gordon, 1992; Damasio et al., 1996, 2004; Farah et al., 1996), functional (Martin et al., 1996; Grabowski et al., 1998; Damasio et al., 2004; Hauk et al., 2004; Pulvermüller et al., 2009), and theoretical studies (Farah and McClelland, 1991; McClelland and Rogers, 2003). These studies show that far from being a locally restricted phenomenon, semantic processing involves the participation of several and highly distributed regions, which are proposed to contain specific parts (nodes) of a whole concept (Damasio and Damasio, 1994; Tranel et al., 1997a; Pulvermüller et al., 2009). As pointed out originally by Wernicke (1874/1977, 1900), the comprehension of meaning implies the recruitment of distributed neuroanatomical areas to create a unified and coherent significance. He proposed that the concept is formed by the total sum of the memory images associated with, say, a particular object. This meant that in order to comprehend meaning, a rapid temporal association had to be made between the acoustic material and the various sensory memory images representing the concept itself (Gage and Hickok, 2005). In neurophysiological terms, a temporal binding of the electrical activity representing local characteristics of whole information is required in order to construct and recognize the total pattern representing the information to be identified (Varela et al., 2001). Our results are in agreement with this proposal, showing that the establishment of functional couplings across distant cortical areas is a prominent phenomenon occurring during semantic analysis, and that these functional interactions are strengthened during the cognitive requirements associated with the recognition and categorization of specific information. These findings predict that during natural language processing, the spontaneous creation of referential knowledge across the discourse would enhance the synchronization patterns of contingent lexical material, which can be the subject of future studies.

### Conflict of interest statement

The authors declare that the research was conducted in the absence of any commercial or financial relationships that could be construed as a potential conflict of interest.
